# Absence of holistic sexual health understandings among men and women in deprived areas of Scotland: qualitative study

**DOI:** 10.1186/s12889-019-6558-y

**Published:** 2019-03-12

**Authors:** Lisa McDaid, Kate Hunt, Lesley McMillan, Siân Russell, Dona Milne, Rosie Ilett, Karen Lorimer

**Affiliations:** 1 MRC/CSO Social and Public Health Sciences Unit, University of Glasgow, 200 Renfield Street, Glasgow, G2 3AX UK; 2 Institute for Social Marketing, University of Stirling, Stirling, UK; 3 Department of Sociology and Social Policy, Glasgow Caledonian University, Glasgow, UK; 4 Institute of Health & Society, Newcastle University, Newcastle upon Tyne, UK; 5 NHS Fife, Kirkcaldy, UK; 6 Independent Research Consultant, Glasgow, UK; 7 School of Health & Life Sciences, Glasgow Caledonian University, Glasgow, UK

**Keywords:** Holistic sexual health, Wellbeing, Heterosexual, Health improvement, Deprivation, Relationships, Coercion, Inter-personal violence

## Abstract

**Background:**

There is a growing evidence base for the need for a holistic approach to sexual health improvement, but the challenges for realising this in the ‘real world’ may be harder in some communities than others. We examined sexual health understandings and behaviours among adult men and women in deprived areas of Scotland.

**Methods:**

Thematic analysis, using the constant comparative method, of qualitative, semi-structured in-depth interviews with 19 men and 16 women aged 18–40 years from the most deprived areas of Glasgow, Edinburgh, Dundee, and three Highland towns.

**Results:**

Even though most had been shown images designed to facilitate discussion about sexual consent and verbal/physical abuse, when first asked, participants overwhelmingly equated ‘sexual health’ with the avoidance of sexually transmitted infections (STIs) and pregnancy. Most of the women interviewed went on to locate their accounts of sexual health within a broader, social account of relationships that in an ideal world, in contrast with their everyday lives, were based on respect and freedom from violence. They expressed desires for more positive relationships, based on open communication and trust, choice and freedom from coercion. A few men did accept a broader definition of sexual health, but others actively resisted it and placed the onus to enact choices and freedom from coercion on women rather than men.

**Conclusions:**

In the first UK study to examine understandings of holistic sexual health among adults living in deprived areas, we found a disjuncture between men and women. These findings suggest that, as a society, we are failing to equip people to enhance their own, and others’, sexual health and wellbeing in its broadest sense. New efforts to emphasise the breadth of sexual health are required, but addressing these complex issues, especially where there are negative underlying gender norms to challenge, will require multi-level interventions targeting individual, community and system levels.

**Electronic supplementary material:**

The online version of this article (10.1186/s12889-019-6558-y) contains supplementary material, which is available to authorized users.

## Background

The World Health Organisation (WHO) frames sexual health as “a state of physical, mental and social well-being in relation to sexuality. It requires a positive and respectful approach to sexuality and sexual relationships, as well as the possibility of having pleasurable and safe sexual experiences, free of coercion, discrimination and violence” [1]. The principles of this broadly accepted definition are reflected in the Department of Health’s Framework for Sexual Health Promotion in England and the Scottish Government’s Sexual Health and Blood Borne Virus Framework (2015–2020), which both aim to deliver on positive sexual health at the individual and societal level [2, 3]. For example, the outcome-based Scottish Government’s Sexual Health and Blood Borne Virus Framework contains an outcome that ‘sexual relationships are free from coercion and harm’, as well as one that sets out to achieve ‘fewer newly acquired blood borne virus and sexually transmitted infections’.

There is growing evidence of the need for a ‘holistic’ approach to sexual health, but less evidence of how to deliver this [4]. Sexual violence is associated with HIV, sexually transmitted infections (STIs) and unplanned pregnancy and non-volitional sex and sexual dysfunction are known to have negative effects on mental health [4]. This highlights the importance of extending our understanding of sexual health beyond the traditional biomedical outcomes of STIs and unplanned pregnancy and the individual risk behaviours, such as the non-use of condoms. Yet, very few studies have explored how health improvement efforts can capitalise on or seek to improve positive sexual health [5]. In turn, there are multiple risk factors for sexual violence, many of which derive from the community and societal levels, and there is evidence that peer norms can support such violence [6]. Again, there is limited evidence of effective strategies for the prevention of sexual violence, and despite recognition of the need to intervene at the community and societal level, very few studies have addressed risk factors beyond the individual level [7]. Furthermore, the challenges of enacting a ‘holistic’ approach to sexual health in the ‘real world’ may be much harder in some communities than others.

As noted above peer norms can negatively influence attitudes and behaviour [6]. The social construction of masculinity in our society – the attitudes, behaviours and characteristics that are associated with being male – impact on men’s (and women’s) relationships and health [8–13]. Dominant forms of masculinity are associated with toughness, aggression and excessive risk-taking, plentiful sexual encounters, and sexual risk-taking behaviour (for example, the non-use of condoms) [9–14]. Evidence focussed on men from areas of high deprivation is limited, but relationships between ‘masculinity’ and ‘health’ are mediated through other aspects of identity and status, such as social class and ethnicity [15, 16]. Previous research has demonstrated conventional beliefs about masculinity among older adolescent and young adult males are associated with poor sexual health [17, 18]. However, the relationship between masculinities and deprivation is complicated [8, 11, 18, 19]. For example, in inner-city areas of deprivation in the North of England, some young men did engage in narratives of respecting their female sexual partners, [20] and protective contraceptive practices were evident among incarcerated men (aged 16–20) interviewed within Scottish prisons, despite many displaying dominant masculine attitudes [21]. Clear trends exist in relation to associations between low socio-economic status (SES) and poor sexual health, [22, 23] but further work is required to explore how masculinity is associated with these in adulthood. Locally, two Scottish Government-commissioned research projects noted associations between socio-economic deprivation and high risk sexual behaviours, despite no knowledge gaps in sexual health, [24] as well as negative attitudes towards women (such as their views about women who carried or used condoms) among men from lower socio-economic groups [25]. While policy efforts seek to impact on such inequalities, [2, 3] our review of literature on masculinities, sexual health, gender and risk taking found only one study focused on the sexual health of adult heterosexual men [26]. Its focus on HIV prevention in African American men limited its generalisability or transferability to the UK context [26]. This, combined with the limited evidence on interventions to improve positive sexual health and sexual violence more broadly discussed above suggests a significant knowledge gap [6, 7].

The Deprivation, Masculinities and Sexual Health (DeMaSH) study aimed to address this knowledge gap and explore sexual health understandings using a masculinities framework to inform intervention development [27]. We sought to bring together three key areas of research: masculinities, to be cognisant of the social context in which sexual relationships are enacted; deprivation, recognising the existing associations between low socio-economic status and negative sexual health experiences; and social and gender relations, given the interplay between women’s and men’s experiences. The findings presented below demonstrate narrow understandings of sexual health among our participants and highlight the scale of the challenge to improving sexual health in its broadest sense in these communities.

## Methods

Nineteen men and 16 women aged 18–40 years from the most deprived areas of Glasgow, Edinburgh, Dundee, and three Highland towns, were recruited to take part in semi-structured, one-to-one interviews. Areas classed as the 10% most deprived were identified using the Scottish Index of Multiple Deprivation (SIMD) interactive mapping tool (http://www.gov.scot/Topics/Statistics/SIMD) and purposively sampled. Multiple recruitment methods were used, including: flyers, postcards, a website and a QR code which could be scanned to direct potential participants to the study website, a short SMS number for people to text, mobile phone number, and an email address; and additionally, talks in community settings, approaches to potential participants in pubs and other public locations, and via gatekeepers within community organisations. All participants were unknown to the interviewer at the time of recruitment, were provided with study information sheets detailing the nature of the study and process involved and gave written informed consent.

The topic guide (Additional file 1) covered perceptions of the local area, including experiences of being a man/woman living there, and sexual health understandings, knowledge and behaviours. Visual prompts on sexual consent and verbal and physical abuse were used to facilitate discussion of these more sensitive topics [27]. The images chosen focused specifically on male perpetrated gender-based violence, including sexual violence. Although we fully recognise that men can be victims as well as perpetrators of such violence, [28] it is more frequently the other way around, and the very premise of this study was to further explore negative attitudes that men living in some of the most socio-economically deprived areas of Scotland held towards women [25]. The images were discussed at length by the project team and Study Advisory Group and chosen to prompt discussion on how prevailing constructions of masculinity in these areas are related to sexual health attitudes and behaviours in adult heterosexual men. The prompts included images of: A) three young men carrying an unconscious, partially undressed woman, allegedly (according to the strapline on the image) after she had been given the ‘date rape’ drug Rohypnol; B) a Police Scotland campaign on alcohol and consent depicting a rugby player, with the caption ‘I’m the kind of guy who doesn’t have sex with a girl when she’s too drunk. Are you?’; C) three still images taken from a Home Office 2012 television campaign advert highlighting the issue of rape within the context of a relationship; D) a depiction of domestic violence with man’s fist looming over a women with a bruised face, with the caption ‘Women deserve equal rights and lefts’; and E) an image depicting the impact of verbal abuse, which showed a fist emerging from a man’s mouth pulling a woman’s hair, with the caption ‘verbal abuse can be just as horrific’.

The interviewer (SR, a female post-doctoral researcher, trained in qualitative research methods) asked participants a series of questions including, ‘What do you think sexual health means?’, ‘How would you define sexual health?’, and/or ‘When I say sexual health, what pops into your mind?’ The nature and flow of the interview resulted in the majority of men and women being asked these questions after being shown the images (described above) relating to sexual consent and verbal and physical abuse. Others were specifically prompted to consider whether these images, or the discussion prompted by the images, were related to sexual health. In this paper, themes are derived from the responses to these questions. The interview guide, including the images, were piloted tested.

Data collection proceeded first with interviews with men, and later with women, allowing anonymised extracts of men’s accounts of sexual health to be presented to women as vignettes for them to respond to in relation to their own experiences. Interviews lasted between 45 min and 2 h (1 h on average) and were conducted in private in community settings. Interviews were audio-recorded (with two exceptions) transcribed verbatim, read repeatedly and coded by SR and KL for analysis. Data management was assisted by using QSR NVivo10. For this paper, coded data were reviewed, analysed and summarised by LMcD, DM and RI and thematic analysis was undertaken, using the constant comparative method to ensure that the analysis represents all perspectives and negative (‘deviant’) cases were explored. When extracts from interviews are used to illustrate analytical points, they are attributed to participants using pseudonyms, together with their age range and current relationship status. We used a gender relations perspective to compare and contrast the everyday lived experiences of men and women, as well as views of self and each other [29].

Ethical approval was granted by the School of Health & Life Sciences, Psychology and Allied Health Sciences Ethics Committee, Glasgow Caledonian University (Reference No. PSYEC12/APP202).

## Results

Of the 35 interviews conducted, we have data on where in the interview the questions on understandings of sexual health were asked for 32 participants (interviews for two women were not audio-recorded and the information is not identifiable in the notes on these interviews, and one participant was not asked the question). Of the 32 who were asked about their understandings of sexual health, 27 participants (and all of the women) were asked after seeing all of the images described above; 10 were specifically prompted to consider whether these images were related to sexual health (e.g., ‘Thinking about these images, what does sexual health mean…?).

Given that participants were specifically prompted to consider sexual consent and verbal and physical abuse as dimensions of sexual health, we could have expected participants’ definitions of sexual health to broadly reflect this. Instead, their accounts showed marked differences in the extent to which men’s and women’s understandings of ‘sexual health’ moved beyond narrowly-defined definitions of the prevention of STIs and pregnancy, to those which encompass broader physical, mental and social wellbeing as defined by the WHO [1]. We compare and contrast understandings by gender in relation to these dimensions below.

### Sexual health as limited to physical prevention of STIs and pregnancy

Even though most had been shown images designed to facilitate discussion about sexual consent and verbal and physical abuse as described above, when first asked about ‘sexual health’, male participants overwhelmingly equated this term with the avoidance of STIs and pregnancy:

“***OK. When I say the phrase sexual health, what pops into your mind?***

R: Er, sexual health? Contraception obviously and then hygiene and um, that’s what pops into my mind.

***OK so how about things like STIs?***

R: Yeah that’s why I say contraception and having that in mind, STDs and HIV and um, the other one.”

(Shane, aged 26–35 years, open relationship)

Shane was asked this question before he was shown the images, but other men offered similar definitions, even after seeing the images:

“I thought it was mainly like about condoms and being careful, no’ catching STIs, that’s just what I thought.” (Daniel, aged 36+ years, single)

Most of the women interviewed also provided similar accounts of their understandings of ‘sexual health’ as being focused on protecting the physical body from STIs and pregnancy:

“Sexual health is taking care of yourself, sexually, all your sexual organs, being checked, going for tests, using protection – not just the pill or, but use condoms.” (Jade, aged 36+ years, single)

Sexual health? Just obviously being aware, you know, if you have unprotected sex, you know, you’re gonna [going to], you could catch something and you know you need to be, you know, look out for yourself, be aware, you know if you have unprotected, you know, you can catch something, and… yeah, and just…(Leanne, aged 18–25 years, single)

Both Jade and Leanne were shown the prompt images before being asked about sexual health, but despite this restricted themselves to narrow discussions of sexual health. However, most female participants, as illustrated below, also offered accounts of sexual health that went beyond this initial focus on the prevention of STIs and pregnancy.

### Broader accounts of sexual health

Although the WHO definition of sexual health and wellbeing specifically refers to mental wellbeing, few participants offered unprompted accounts of sexual health that explicitly related to this. Megan was an exception:

“Sexual health means, like, all round. Like, I dunno [don’t know], mental, health around it, physical health. Aye.

**So when you say mental health, what sorts of things do you mean?**

Well, like, your mental attitude towards sex. So, like, mebbe [maybe] someone will see, think it’s all right to go out, and if a woman says no, d’you know what I mean? That’s still sexual health. Cos sexually, his mind’s no’ healthy. So that’s sexual health as well as physical, whether you’ve got an infection.

**You are the first person who’s gone straight out with that. A lot of people go straight for the STIs, despite having discussed all of this. So why d’you think so many people don’t, like, respond like you have? So many people just think STIs rather than –**

Because that’s what they get taught, when they hear the word sexual health, it’s about putting a condom on and taking these tablets to get rid of this. Rather than the mental aspect tae [to] sexual health.

(Megan, aged 18–25 years, long term relationship)

More often, it was the relationship context that was raised in the discussion and appeared to be particularly important to the women interviewed:

“…You need to be happy in your relationship. So there is like a psychological side of it as well, as well as your STIs and whatever.” (Kayleigh, aged 18–25 years, Single)

“And just making sure, like, when youse [you] are having sex that... do you know, it's what youse [you] both want an'... is that what you...? Yeah? Yeah, just aboot [about], like, two couples, if that's what they're doing then they talk about it and, you know, there's things in place for them, they're both happy wi' [with] what's going on an' they have a good relationship where they can talk tae [to] one another an' it's all happy.” (Shannon, aged 26–35 years, separated)

Kayleigh and Shannon were specifically prompted to consider whether the images they had been shown were related to sexual health, but the majority of the women we interviewed located their accounts of sexual health within a broader, social account of relationships, without prompting. This social account of relationships was one ideally based on respect and a freedom from violence:

**“** ***So what do you think sexual health means?***

I think for me it’s just about being safe.

***How do you mean?***

Safe against STDs and safe in relationships.

***So when you say safe in relationships ….?***

Safe in relationships with regards to sexual violence. And just because people seem… I think it’s almost ok for younger guys and younger men to treat people, to treat women like they are pieces of meat. But that’s through things like media, things like porn. Things like films, music, anything really.” (Angela, aged 26–35 years, single)

Even though all of the women were asked about sexual health after having seen the prompt images, their reported experiences of domestic violence were almost always more experiential and personal than they were for men. Ten women described experiencing or witnessing gender-based violence of some form, with six of them describing surviving abusive relationships or experiences, including sustained and often coercive and controlling relationships with violence over months or years. Some described these as “hell”, or talked of how this made them reach “rock-bottom” as their self-esteem was undermined.

Women talked of the need not just to have respect from a partner, but also to have respect for themselves. They expressed desires for more positive relationships, based on open communication and trust, choice and freedom from coercion:

“Just think people forcing you tae [to] have sex should be, like, tae [to] force you, in a relationship, if you don’t want tae [to] have sex, you don’t have sex. You don’t get forced.” (Chloe, aged 18–25 years, long-term relationship)

In contrast, just four of the 15 men who were asked about sexual health after being shown the prompt images offered accounts of sexual health that went beyond initial focus on the prevention of STIs and pregnancy:

“Sexual health? Just being safe and being clean, not having STIs, not being, like a lot of these pictures, being bad towards women or forcing yourself upon them or being inappropriate towards them. Yeah, sexual health – that could mean a various number of things, I think, yeah.” (Ryan, aged 36+ years, long-term relationship)

“I don’t think it should be any of that in relationships. I think it should be equal either way, shouldn’t it? Know what I mean? No-one should be – whether it’s her getting hit or him getting hit, or her getting abused, him getting abused. It just shouldn’t happen, should it? Everybody should be equal. I would say, that’s it. Everybody should be equal. Shouldn’t be any of that.” (Scott, aged 36+ years, single)

When explicitly, or further, prompted to consider a broader definition of sexual health by the interviewer, a few men did accept it:

“No, obviously that’s mental health obviously maist [most] of these [referring to images]. Obviously that yin [one], well a’ o [all of] them pretty much so…

***Interviewer: So sexual – but you don’t think sexual health can be to do with your mental health at the same time?***

Obviously it can mess wi’ [with] your head as well obviously.” (Connor, aged 18–25 years, single)

However, in doing so, a few men still appeared to interpret mental and social wellbeing in terms of the ultimate impact on physical health to some extent:

“[Image D – domestic violence] just… I mean, it’s no’ [not] just aboot [about] sex, is it? It’s just unhealthy relationship, unhealthy lifestyle for that lassie [girl/woman] that’s tae [to] be living you know?

***Interviewer: Yeah. But in terms of her sexual health, do you think she has any choice over where she has sex, the type of sex she has?***

Nah. Definitely no’. I’m sure her sexual health will be non-existent probably, do you know what I mean? I mean, he’ll be taking what he wants when he wants by the looks o’ [of] that photograph. […] I mean, he could be oot [out] sleeping wi’ [with] other women and passing on all sorts. I don’t’ think the boy’s gonnae be putting condoms on and that, ‘cause I’m sure he’ll be making sure she’s no’ sleeping wi’ anybody else…” (Ally, aged 36+ years, single)

***“Do you think any of these relate to sexual health?***

Maybe... I don't know. Yeah. Maybe [Image A – Rohypnol]. Obviously because if you get spiked wi' [with] rohypnol and you're not in control of what's happening or anything like that so, I mean, you can't turn round and say “Oh, you need tae [to] put a condom on” or, you know, anything like that. So, I mean, [Image A – Rohypnol] would be... again, that's me coming back tae [to] the safe sex type thing.” (Josh, aged 26–35 years, long-term relationship)

And in some cases, it was the potential impact on their own physical sexual health that was of concern:

“Oh wait a minute, what does this say *'I'm not the kind of guy to have sex with a girl when she's too drunk'*. Yeah that's related to sexual health as well, that's what I would think you know. If [Image B - Police Scotland campaign on alcohol and consent] like if you're too drunk, I want to think maybe you did this more often. And if you're going to have sex with me when you're in that state then how many guys have you had sex with in that state. So the more likely to have an STI [if] you're the kind of girl who would have sex with me when you are too drunk. So I would worry about my sexual health in that situation.” (Shane, aged 26–35 years, open relationship)

Some men remained actively resistant to broader definitions of sexual health, despite specific prompting:

**“Interviewer:** ***So do you think these images are connected to sexual health and how so?***

Sexual health tae [to] me, in probably my ignorance, doesn’t involve domestic violence, physical or mental, to be honest. I think more sexual health as the actual sex and/or the STIs or the physical relationship, I suppose would be the best way for me tae [to] put it, rather than the relationship side of it, if that makes sense? […] I suppose you wouldnae [wouldn’t] want tae [to] have sex wi’ [with] someone that’s slagging you off all the time or beating you up and, obviously, that can have an effect on future partners. But I would still say the definition, tae [to] me, of sexual health is the physical relationship, the sex or the kissing or the oral sex or whatever and the STIs that come from that or… rather than the domestic violence which, tae [to]me, is a separate category.” (Luke, aged 18–25 years, single)

***“So what do you think sexual health means?***

Well, obviously your sexually transmitted diseases and stuff like that. I don't really know, tae [to] be honest. Obviously your STDs and stuff like that and obviously your transmitted diseases but... other than that... […] Because they're all tae [to] do wi' [with], you know, like people forcing sex on people and... (pause) it's all tae [to] do wi' [with] kind o' relationships and between men and women and... (pause) I mean, tae [to] be honest, I can see the point o' a' [of all] this kind o' [of] stuff being here but I didn't think that there would be anything like that.

***You mean [Image D – domestic violence] and [Image E – verbal abuse]?***

Yeah, image D and E, yeah. I wouldnae [wouldn’t] really think that they had much tae [to] do wi' [with] sexual health. And you think o' [of] health, you automatically think o' [of] disease, stuff like that. Not these kind o' [of] issues.” (Thomas, aged 26–35 years, long-term relationship)

Furthermore, when presented with the images of domestic violence, a few men talked about women having the ‘choice’ to leave or walk away from situations in which they were threatened with violence, with the onus clearly on the woman to take action:

***“So in terms of these relationships, here, the [Image E – verbal abuse] and [Image D – domestic violence], what kind of choices do you think the women in those relationships had in terms of when they had sex and things like that?***

I don’t know? They probably don’t have a choice. There’s always a choice to get oot [out] it though.” (Jim, aged 18–25 years, single)

“…they always have sort o’ the option tae [to]… they always have the option tae [to] walk away. I know that sometimes that it’s not gonnae [going to] be as easy as just walking away ‘cause, you know, they might love the person or, you know, they believe that they can change him…” (Josh, aged 26–35 years, long-term relationship)

This was the case even though many men (including Luke and Thomas above) spoke in some way about witnessing or knowing about the experience of domestic abuse. Few men talked about experiencing or perpetrating abuse (and they may have been disinclined to do so to a female interviewer), but eight did talk about witnessing it. For example, Rab recalled a vivid childhood memory of hearing his mother “getting battered off the walls”. He went on to say:

“…sometimes you still get the images and things like that and you just freak oot [out] and you just cannae [cannot] get rid o’ [of] the images and you see what happened to your family, and that’s why I’m against it [violence against women], d’you what I mean? I just don’t trust guys.” (Rab, aged 18-25 years, single).

While this quote suggests that Rab judges such behaviour to be unacceptable or needing to be challenged, this was rare in other men’s accounts, even when discussing rape, as illustrated by an extract from Josh’s interview:

“It might just be that one time when she’s not feeling it [does not want to have sex] and he’s putting more pressure and pressure on her. If anything was tae [to] happen then it would be rape and, again, *she can walk away*.” (Josh, aged 26–35 years, long-term relationship, emphasis added)

Here, Josh clearly places responsibility for avoiding rape with women rather than suggesting that men should take responsibility for this coercive behaviour.

## Discussion

This is the first UK study to examine understandings of holistic sexual health among adult men and women living in deprived areas. Our gender comparative analysis allowed us to interpret and assess the implications of men’s understandings compared with those of women. It is striking that despite being shown a series of images related to sexual consent and violence, when first asked, most participants (and in particular the men we interviewed) overwhelmingly equated sexual health solely with the avoidance of STIs and pregnancy. In response in part to the images we used, most women were able to locate their accounts of sexual health within a broader, social context and a desire for relationships based on respect and free from violence. In contrast, few men accepted a broader definition of sexual health, even when prompted, and instead they privileged narrow, physical aspects of sexual health. Our findings offer parallels with the discourse that sets, and often fails to question, reproductive and sexual health as a ‘women’s’ issue and research (with predominantly younger age groups) demonstrating how gendered power relations and dominant masculine discourses serve as a barrier to men taking responsibility for (or talking about) their own sexual health [20, 29–31].

Most worryingly, men reframed the onus to enact choices and freedom from coercion on women rather than men. This is similar to findings with younger people in England in which young women reported expectations of being pressured to have sex in relationships and women who were blamed for sexual exploitation and violence [32]. This exposes a perpetuation of the ‘just say no’ discourse which typically suggests that women should bear responsibility for consent by clearly and directly declaring ‘no’. This discourse has long been criticised for: ignoring the complexities inherent in how every day refusals are performed; denying the role of cultural norms, power, and sexual scripts; and ultimately sanctioning ‘miscommunication’ as an excuse for denying responsibility for coercion or rape [33–35]. Such gender imbalance is consistently reported and of global concern [36]. Our findings, in conjunction with the literature, suggest that as a society we are not equipping people to enhance their own, and others’, sexual health and wellbeing in its broadest sense. This is not about promoting sexual health per se, but about recognising the need to challenge the lack of recognition for countering coercion and sexual violence as a core part of maintaining sexual health and wellbeing.

### Strengths and limitations

Key strengths of the study were its originality and the gender comparative approach, which highlights the potential impact of men’s attitudes and understandings on women, and our focus on people aged 18–40 years, when most research elsewhere has focused on adolescent populations only. Presenting women with men’s (anonymised) accounts of their attitudes to women could have influenced the women’s accounts, but participants were asked for their own views first before being offered these vignettes. We believe that their own life experiences influenced them more than hearing what the men said, and perhaps hearing men’s views created a space that allowed them to reflect and draw upon their own experiences. As a result, they may have been more open to holistic understandings of sexual health. To fulfil the aims of the DeMASH study, data collection was restricted to Scotland and to areas of high deprivation. Although parallels can be seen elsewhere, [29, 30, 32, 36] further research should examine the extent to which our findings are reflected across a wider range of geographical and socio-economic areas, to investigate whether the lack of broader sexual health understandings that we have observed here need to be addressed more generally in our society. Further research is also required to examine male-to-male sexual assault, which was beyond the scope of this study. Finally, that our participants were asked to define sexual health after being shown the images on sexual consent demonstrated that even when sensitised to issues of consent, violence and wellbeing, men overwhelmingly still did not equate these with sexual health. If men cannot speak of or relate to broader definitions of sexual health in this context, we wonder what circumstances would allow for it.

### Implications

Resistance among policy makers and practitioners to a broader approach to sexual health has been noted [4] and our research suggests such resistance could extend well beyond the policy arena to the general public. Although holistic sexual health and wellbeing is now core to policy in the UK, [2, 3] sexual health has primarily been equated with the avoidance of STIs and pregnancy for far longer and very few adult sexual health intervention studies have gone beyond the traditional, biomedical outcomes of STIs and unplanned pregnancy [4, 5]. It is likely that current resource constraints will now threaten efforts to promote a more holistic approach to sexual health and wellbeing in clinical settings and sexual health services. We need to find new ways to emphasise ‘holistic’ sexual health and provide support to people that goes beyond the traditional provision of clinical testing and treatment services, and ensure that these efforts do not bypass the key audiences by not speaking to their lived experience. The continued narrowness of understandings of sexual health and the disjuncture between men’s and women’s understandings in the communities included in our research highlights the depth of the challenge faced in improving adult sexual health and wellbeing in its broadest sense. Our data can inform work to promote sexual relationships that are free from harm and coercion (one of the five core outcomes of the Scottish Government’s Sexual Health and BBV Framework) [3].

## Conclusions

The findings, in conjunction with the wider literature, demonstrate the importance of developing ways to support positive relationships, enabling men and women to better understand each other. There is a clear need to emphasise the importance of equal and respectful relationships to wellbeing, but practitioners, policy makers and researchers must do so in ways that meet the needs of people within communities at greatest risk of poor (holistic) sexual health. Individual-level interventions have an important part to play, but it is unlikely that they alone will impact on these complex issues, which are in part driven by the intersections between gender, poverty, and violence in some communities, and supported by strongly negative peer and cultural norms [27]. Instead, addressing these complex issues and challenging norms will require multi-level interventions that target the individual-, peer- and community-level [37, 38]. Such efforts are likely to be best introduced early and reinforced across the lifecourse, and most importantly, co-produced with the communities in question as part of broader approaches to education, health and wellbeing.

## Additional file

Additional file 1: DeMASH Topic Schedule. Example interview schedule from the DeMASH study. (PDF 644 kb)

## Abbreviations

DeMASH Study: Deprivation, Masculinities and Sexual Health study
SES: Socio-economic status
SIMD: Scottish Index of Multiple Deprivation
STIs: Sexually transmitted infections
WHO: World Health Organisation
