# Supplementary material for: Absence of holistic sexual health understandings among men and women in deprived areas of Scotland: qualitative study
Source: BMC Public Health. 2019 Mar 12;19:299. doi: 10.1186/s12889-019-6558-y (PMC6417029; doi:10.1186/s12889-019-6558-y)
Supplement: Supplementary file 1 — DeMASH Topic Schedule. Example interview schedule from the DeMASH study. (PDF 644 kb) [file 12889_2019_6558_MOESM1_ESM.pdf]

Glasgow Caledonian University

**INTRODUCTIONS:-**

*[introductory conversation]*

**PART 1: WHERE YOU LIVE:-**

- **So what's it like to live around here?**
  - good/bad aspects – and why
- **What's it like for women around here?**
  - Why might that be?
  - Any differences

**PART 2: BEING A MAN**

- **Here is a selection of images of men – some are famous, some are not.**
  - **Image Card 1 [Images A - B: - Chewin' the fat NEDs + Begbie]**
  - **Image card 2 [Images C - D:- Andy Murray + Thomas Hitzlsperger]**
  - **Image card 3 [Images E - F:- Kevin Bridges + Hardeep Singh Kohli]**
  - **Image card 4 [Images G - H:- Hipster + Robert Preston]**
    - What do you think about these men? | Any of these guys fit in here?
    - So, what is a typical man from [area] like?
    - Which of these images do you identify with?

- **Here is a selection of images of women– some are famous, some are not**
- **Image card 5 [Images A - B: - Stereotypical NED + Subtle NED]**
- **Image Card 6 [Images C - D:- Rebecca Adlington + Jessica Ennis]**
- **Image card 7 [Images E - F:- Paris Hilton + Shami Chakrabarti]**
- **Image card 8 [Images G - H:- Housewife + 'Gold digger']**
    - What do you think about these women?
    - Which of these women seem like a typical woman from where you live?
    - So let's play snog, marry or avoid... Why / expand etc.

- **How do men act around women in [area]?**
    - Would you act differently with women than with men?
    - Are there any things which a man *shouldn't* do around women?
    - How about different kinds of women? **[refer to images]**
- **How do women act around men?**
    - Are there any things women shouldn't do around men?

## PART 3: SEXUAL HEALTH/BEHAVIOURS

- ☐ **How did you learn about sex?**
  - School / parents / friends?
  - Magazines / the Sun /The Star
  - Porn?
    - How about boys growing-up today?

- ☐ **How about women?**
  - Would they have learned about sex in the same way?
    - What about girls growing up today?

- ☐ **What does sex mean to you?**
  - How important is sex in your lives?
  - Why do you think that is / can you expand..?
  - What role might **porn** play?

- ☐ **What does sex mean to women?**
  - Probes as above

- ☐ **Condoms. What do you think about them?**
  - Do you use them?
  - How about women carrying condoms?

## PART 4: CONSENT, ABUSE, ALCOHOL

I'm going to show you a few images with captions, which have been taken from the Internet. I'd like for you to look at the image and tell me what you think.

**[Image card 9:- Image A, Rohypnol]**

- ☐ **What do you think of this image? | What's happening here?**
    - What *should* be happening here?
- ☐ **Have you seen something like this happen? | What would you do if you saw this happening?**
- ☐ **It calls women 'teasing bitches'. What do you think about that?**
- ☐ **What role has alcohol played?**
    - Does alcohol play a role in your sex life? | Do you drink to pull?
    - Do you need it? | How does drinking make you feel?
    - Where do you tend to drink?

**[Image card 10:- Image B, 'Too Drunk"]**

- ☐ This image is from an ad campaign. What do you think of it?
    - What do you think of the ad?
    - **The ad states "too drunk". What do you think 'too drunk means?**
    - **How would you tell?**

**[Image card 11:- Image C, "Consent in relationships"]**

- ☐ **This is a series of images of a young couple. Neither of them are intoxicated**
    - What is happening in this scene?

**[Image card 12:- Image D, physical abuse]**

- ☐ What do you think of the image?

**[Image card 13:- Image E, Verbal abuse]**

- ☐ What do you think of this image?

**Considering what we've just discussed, what do you think 'sexual health' means?**
